# Clinical Characteristics and 30-Day Outcomes of Intermittent Hemodialysis for Acute Kidney Injury in an African Intensive Care Unit

**DOI:** 10.1155/2016/2015251

**Published:** 2016-03-03

**Authors:** Arthur Kwizera, Janat Tumukunde, Lameck Ssemogerere, Emmanuel Ayebale, Peter Agaba, Jamali Yakubu, Aggrey Lubikire, Mary Nabukenya, Robert Kalyesubula

**Affiliations:** ^1^Department of Anaesthesia, Makerere University, P.O. Box 7072, Kampala, Uganda; ^2^Dialysis and Intensive Care Unit, International Hospital Kampala, P.O. Box 8177, Kampala, Uganda; ^3^Departments of Physiology and Internal Medicine Nephrology Unit, Makerere University, P.O. Box 7072, Kampala, Uganda

## Abstract

*Introduction*. Acute kidney injury (AKI) is a common occurrence in the intensive care unit (ICU). Studies have looked at outcomes of renal replacement therapy using intermittent haemodialysis (IHD) in ICUs with varying results. Little is known about the outcomes of using IHD in resource-limited settings where continuous renal replacement therapy (CRRT) is limited. We sought to determine outcomes of IHD among critically ill patients admitted to a low-income country ICU.* Methods*. A retrospective review of patient records was conducted. Patients admitted to the ICU who underwent IHD for AKI were included in the study. Patients' demographic and clinical characteristics, cause of AKI, laboratory parameters, haemodialysis characteristics, and survival were interpreted and analyzed. Primary outcome was mortality.* Results*. Of 62 patients, 40 had complete records. Median age of patients was 38.5 years. Etiologic diagnoses associated with AKI included sepsis, malaria, and ARDS. Mortality was 52.5%. APACHE II (OR 4.550; 95% CI 1.2–17.5, *p* = 0.028), mechanical ventilation (OR 13.063; 95% CI 2.3–72, *p* = 0.003), and need for vasopressors (OR 16.8; 95% CI 3.4–82.6, *p* = 0.001) had statistically significant association with mortality.* Conclusion*. IHD may be a feasible alternative for RRT in critically ill haemodynamically stable patients in low resource settings where CRRT may not be available.

## 1. Introduction

Acute kidney injury (AKI) occurs in 5.7–24% of intensive care unit (ICU) patients [1]. It is commonly associated with multiorgan failure, preexisting renal disease, sepsis, and renal hypoperfusion. In addition to morbidity, AKI is also a common cause of increased length of stay and increased costs of healthcare. Mortality of patients with AKI in ICU ranges from 46.8% to 60% and use of vasopressors, mechanical ventilation, and shock (septic and cardiogenic) are some common independent predictors of mortality [1–3]. The management of AKI ranges from conservative (including etiologic management, hemodynamic support, maintaining fluid and electrolyte balance, avoiding nephrotoxic drugs, and appropriate drug dosing for level of glomerular filtration rate) to renal replacement therapy (RRT) [4]. RRT includes peritoneal hemodialysis (PD), IHD, or CRRT. The preferred choice for RRT among peritoneal dialysis (PD), intermittent hemodialysis (IHD), and continuous renal replacement therapy (CRRT) remains unresolved despite several randomized controlled trials [5].

In low-income countries, studies from India have previously reported the profile and outcome of AKI in ICUs and one study described treatment characteristics of RRT, therapy modification, and sickness profile [6]. Patients from low-income countries with AKI are quite different from those of developed countries in that they are often younger, have less comorbidities, and are likely to have higher rates of HIV infection [7]. It is therefore important to appreciate the notion that studies of patients with AKI from developed countries may not represent the true picture of what happens in low resource settings. Unfortunately, few to no studies have looked at renal replacement therapies in the ICU.

We aimed to study the patient characteristics, RRT practice of a modified IHD, and the outcome of patients with AKI in an ICU in a low-income country university teaching hospital.

## 2. Materials and Methods

A retrospective study of all consecutive patients over the age of 18 who underwent modified IHD in the ICU between January 2012 and May to December 2014 was performed. International Hospital Kampala is a 100-bed private tertiary teaching hospital in Kampala, Uganda, served by 18 outpatient clinics and serving an accessible population of one million people in Kampala. It has a 10-bed multidisciplinary ICU. Patient demographics, clinical characteristics, APACHE II score, reasons for renal replacement, vasopressor use, mechanical ventilation, and biochemical and hematological parameters at the onset of hemodialysis were noted.

Hemodialysis (HD) was performed using the Gambro AK95 machine. Standard water treatment was used. Dialysate concentrates were commercially purchased. F5 polysulfone dialyzers were used (surface area of the dialyzer was 0.9 m^2^). All the patients were anticoagulated with unfractionated heparin based on clinical risk assessment. The intensive care team (intensivist and nephrologist) decided on ultrafiltration based on clinical risk assessment and hemodynamics. IHD characteristics that included blood flow rate (BFR), dialysate flow rate (DFR), anticoagulation, and ultrafiltration (UF) were reviewed. Major complications were documented. Patient survival was defined as dialysis-free discharge from the ICU. Patient characteristics were shown as percentages, mean ± SD, and/or median where necessary. Microsoft Excel and SPSS version 22 (IMB Corporation, Armonk, New York, USA) were used for data analysis. Analysis of variance was used to compare the mean of various parameters with the outcome variable (survival and nonsurvival) and conclusions on associations were made using statistical tests of *p* < 0.05 as being significant. Multivariate analysis was also done using logistics regressions for selected characteristics against the outcome variable (survival and nonsurvival) and results concluded using unadjusted odds ratios above 1 with *p* < 0.05 signifying existence of associations. Other variables were continuous in nature and were categorized into two groups using median to distinguish the groups.

## 3. Results

Out of 62 patients who underwent dialysis, 40 patients had complete records according to the study protocol. They underwent 192 IHD treatments. Among these, 32 (80%) were male. The median age was 38.5 ± 12 years. Distribution by etiology was as follows: sepsis in 33 patients (82%) and malaria in 14 patients (33%). Twenty-three (57.5%) patients required vasopressor treatment for septic shock (see Table 1).

APACHE II mean score was 24.5 ± 3.7. The median BUN was 44.5 + 7.2 mg/dL, and the mean creatinine level was 6.25 ± 1.8 mg/dL.

Mean serum sodium was 134.9 ± 4.7 mEq/L, pH was 7.1, serum potassium was 5.0 ± 0.3 mEq/L, hemoglobin was 10.4 ± 3.7 g/dL, mean total white cell count was 14.3 ± 4.2 cells/mm^3^, and mean platelet count was 143.2 ± 14.5 cells/mm^3^ (see Table 1).

Dialysis sessions were 192 and majority (61.2%) were for <4 h (Table 2). Mean blood flow rate (BFR) was 264.5 ± 42.5 mL/min and dialysate flow rate (DFR) was 474.9 ± 109.8 mL/min. Heparin used was 281 ± 339 U/h, while mean UF was 242.1 ± 27.3 mL/h.

The major complications during dialysis included hypotension in 9 patients (22.5%) and anemia in 7 (17.5%), but majority of the patients did not have any complication.

Analysis of outcome was done at 30 days from the start of IHD and out of the 40 patients, 19 (47.5%) were alive (survivors) and 21 (57.5%) died (nonsurvivors).

In Tables 3 and 4, comparisons of trends and predictors of survival were done among survivors and nonsurvivors. Two patterns were observed after performing analysis of variance: the mean APACHE II score (27.6) for nonsurvivors was higher than for survivors and mean platelet count for nonsurvivors (104.9) was lower than for the survivors (216). These were statistically significant.

Other predictors for nonsurvival identified were vasopressors and mechanical ventilation (Table 4). The majority *n* (90.5%) of nonsurvivors received mechanical ventilation for ARDS and 85.7% of nonsurvivors had also been treated with vasopressors for septic shock (*p* = 0.01). There was no significant association between age, number of dialysis sessions, serum sodium, and serum potassium, hemoglobin, creatinine, urine output, WBC, pH, UF, PT/INR, and blood flow rate between the two groups. Illnesses such as malaria and other complications like anemia, hypotension, and cardiac problems did not have any significant association with ICU admission outcomes.

At multivariate analysis, APACHE II score, vasopressor use for septic shock, and mechanical ventilation were significantly associated with mortality (see Table 5).

## 4. Discussion

This retrospective study was conducted to characterize and determine outcomes and factors associated with critically ill AKI patients undergoing IHD in a low-income tertiary hospital. Our study found that IHD methods were similar to those reported in the literature [8]. However, our mortality differed somewhat from studies in similar settings and was higher than that reported in higher income countries [9]. Our study population was generally younger than that in HICs and similar to previously described populations in similar settings. This may have contributed to the lower mortality than that observed in the Indian setting [6]. The causes of AKI were in keeping with known etiology; however, malaria sepsis emerged as an unusual cause of AKI in our population, considered to be endemic and therefore conferring active immunity. ARDS/need for mechanical ventilation was also of significant association. This could be because high PEEP levels have been associated with AKI even though the mechanism is not fully understood [10].

IHD in this study was complicated by hypotension. This is not an uncommon complication of IHD among patients with CKD. However, septic shock being treated by vasopressors may have worsened outcomes due to the dialyzable nature of vasopressors used. No cardiac arrests occurred during the study period.

Significant predictors of mortality included ARDs/need for mechanical ventilation and septic shock. Sepsis-induced AKI is prevalent in our population and is a documented cause of mortality [7].

The study population illustrated that appropriate modified practice of IHD could be customized to the clinical needs of patients with AKI in ICU. It helped achieve reasonable clinical outcome in environments with resource constraints. Centers similar to our setting have adopted modifications in their practice to minimize complications [11]. The spectrum of ICU patients developing AKI and the age profile in our patient population were similar to ICU in the developing world. They are younger and more likely to be male. The large proportion of sepsis in our series accounted for the high prevalence of multiorgan failure.

While the choice of modality for RRT varies across centers globally [12], the preferred choice in our setting is IHD. It is commonly used for CKD patients [13]. Peritoneal dialysis (PD) is infrequently used and when so is used to treat AKI in paediatric patients [14]. The choice of RRT is due to CRRT being labor-intensive and expensive [15]. This is compounded by a dearth of ICU beds and dialysis equipment in this setting [16]. The above factors are major limitations to utilizing CRRT in Uganda and other low resource settings. Modifications of conventional IHD such as sustained low efficiency dialysis, short daily dialysis, and isolated ultrafiltration have been shown to achieve satisfactory patient outcomes in developing world ICUs. The retrospective nature of the study limited reviewing other important adverse effects including new onset of infections from access, major bleeding, and transient cardiac arrhythmias. Poor hemodynamic tolerance of IHD was a common problem for patients in the ICU. Hypotension occurred in 22.5% of the IHD sessions in this study. This is more than what was reported in other studies [6, 17]. The salient feature was that the patient survival in our study was similar to that published in developed countries. This fact may indirectly indicate that major adverse effects may not have been missed in our data review.

There is yet no consensus on the timing of initiation of RRT [18–20]. The mean BUN and creatinine of patients in our study reflect the timing of initiation to be reasonably consistent with contemporary practice. Additionally, serum urea at initiation of dialysis has no predictive value on in-hospital mortality in ICU patients with AKI [21].

A recent meta-analysis, which attempted to analyze modified IHD versus CRRT, noted no difference in survival with either modality; however, there was significant heterogeneity in these studies [22, 23].

Mortality among patients treated with RRT was associated with organ dysfunction and comorbidity, and it was approximately 58%. This was also reflected in the Indian study (58% at 30 days) [6]. This is however higher than what has been reported in the Ugandan ICU setting (40.1%) [16].

Our study is the first to report on characteristics of RRT in an African setting; however, it was limited by its retrospective nature, missed patient records, inadequate details of dosage, unclear duration of vasopressor use, and possible underreporting of adverse effects.

## 5. Conclusion

These study findings suggest that IHD may be a technically feasible option for RRT in low resource settings that have limited or no access to CRRT. More work needs to be done to determine the viability of IHD for RRT among patients with AKI in low resource settings.

## Figures and Tables

**Table 1:** Clinical features of patients with acute kidney failure.

*N* = 40	*n* (%)
Gender	
Male	32 (80.0)
Female	8 (20.0)
AKI etiological diagnoses	
Sepsis	33 (82.0)
Cardiac	9 (22.5)
Malaria	14 (33.0)
ARDS	17 (42.5)
TBI	2 (5.0)
Poisoning	1 (2.5)
Organ support	
Mechanical ventilation	27 (67.5)
APACHE II (mean)	24.5 ± 3.7
Vasopressors	23 (57.5)
Enteral feeds	25 (62.0)
Laboratory characteristics	
Creatinine (mmol/L)	6.25 ± 1.8
Sodium (mEq/mL)	134.9 ± 4.7
Potassium (mEq/mL)	5.0 ± 0.3
pH	7.1 ± 0.7
Haemoglobin (g/dL)	10.4 ± 3.7
White blood count (10^3^)	14.3 ± 4.2
Platelets	143.2 ± 14.5

AKI: acute kidney injury; APACHE II: Acute Physiological and Chronic Health Evaluation; ARDS: acute respiratory distress syndrome; TBI: traumatic brain injury.

**Table 2:** Hemodialysis prescription characteristics and outcomes.

*N* = 40	*n* (%)
Total number of dialysis sessions	192
Duration of dialysis (hours)	
<4	118 (61.2)
4–6	30 (17.3)
>6	44 (22.5)
Blood flow rate (mL/min)	264.5 ± 42.5
Heparin (U/hr)	281 ± 39
Ultrafiltration rate	242.1 ± 27
BUN	44.5 ± 7.2
Creatinine (mmol/L)	6.5 ± 1.85
Complications during dialysis	
Anemia	7 (17.5)
Hypotension	9 (22.5)
Hypotension/anemia	2 (5.0)
No complication	22 (55.0)
Outcome	
Alive/discharged (survivors)	19 (47.5)
Dead (nonsurvivors)	21 (52.5)

AKI: acute kidney injury; APACHE II: Acute Physiological and Chronic Health Evaluation.

**Table 3:** Comparison of characteristics of survivors and nonsurvivors with acute renal failure in ICU.

Variable	Survivors	Nonsurvivors	*p* value
APACHE II score	20.9	27.6	0.004
Age (years)	38.9	49.7	0.082
Number of dialysis sessions	5.2	4.5	0.603
Sodium (mEq/mL)	130.0	138.0	0.078
Potassium (mEq/mL)	5.0	5.0	0.989
Hemoglobin (g/dL)	10.3	10.6	0.783
WBC count (10^3^ cells/mm^3^)	15.4	13.3	0.613
PT/INR	1.7	2.3	0.156
Platelet count (cells/mm^3^)	216.0	104.9	0.002
pH	7.16	7.21	0.279
Blood flow rate (mL/min)	270.0	251.0	0.517
UF (liters)	2.01	1.84	0.558
Average duration (hours)	3.63	3.514	0.730
Creatinine (mg/dL)	41.2	65.6	0.781

**Table 4:** Comparison of characteristics of survivors and nonsurvivors with acute renal failure in ICU.

	Survivors	Nonsurvivors	*N* = 40	*X* ^2^	*p* value
	*n* (%)	*n* (%)			
Mechanical ventilation					
Ventilated	8 (42.1)	19 (90.5)	27		
Not ventilated	11 (57.9)	2 (9.5)	13	10.639	0.01
Vasopressors					
Applied	5 (26.3)	18 (85.7)	23		
Not applied	14 (73.7)	3 (14.3)	17	14.401	0.01
ARDS	5 (26.3)	12 (57.1)	17		
Non-ARDS	14 (73.7)	9 (42.9)	23	3.879	0.05
Complications					
Anemia	3 (15.8)	4 (19.0)	7		
Hypotension	2 (10.5)	7 (33.3)	9		
Anemia/hypotension	0 (0.0)	2 (9.5)	2	6.473	0.09
No complication	14 (73.3)	8 (38.2)	22		
Malaria cases	7 (36.8)	7 (33.3)	14		
Nonmalaria cases	12 (63.2)	14 (66.7)	26	1.054	0.86
Cardiac cases	15.5 (3)	6 (6)	9		
Noncardiac cases	84.2 (16)	15 (71.4)	31	0.933	0.34

AKI: acute kidney injury; APACHE II: Acute Physiological and Chronic Health Evaluation.

**Table 5:** Multivariate (risk factors) analysis of the predictors of survival and nonsurvival.

	Nonsurvivors	Survivors	Odds (95% CI)	*p* value
	*n* (%)	*n* (%)		
APACHE II > median (24)	13 (61.9)	5 (26.3)	4.550 (1.181–17.524)	0.028
Mechanical ventilation	19 (90.5)	8 (42.1)	13.063 (2.343–7.818)	0.003
Use of vasopressors	18 (85.7)	5 (26.3)	16.854 (3.416–82.602)	0.001
ARDS			3.733 (0.979–14.222)	0.054
